# Acute Pancreatitis and Diabetic Ketoacidosis: Special Considerations in Diagnosis and Management

**DOI:** 10.1155/crie/7514829

**Published:** 2025-11-11

**Authors:** Amir Babiker, Abdulrahman A. Aldeeri, Ehssan Basha, Ghalyah Aljowaie, Areej Al Sunaid, Mohsen Al Atawi, Ahmad Amer Al Boukai

**Affiliations:** ^1^College of Medicine, King Saud Bin Abdulaziz University for Health Sciences, Ministry of the National Guard Health Affairs, Riyadh, Saudi Arabia; ^2^King Abdullah Specialized Children Hospital, Ministry of the National Guard Health Affairs, Riyadh, Saudi Arabia; ^3^King Abdullah International Medical Research Centre, Ministry of the National Guard Health Affairs, Riyadh, Saudi Arabia; ^4^Department of Genetics, Internal Medicine, College of Medicine, King Saud University, Riyadh, Saudi Arabia; ^5^Department of Radiology, College of Medicine, King Saud University, Riyadh, Saudi Arabia

**Keywords:** acute pancreatitis, amylase, diabetic ketoacidosis, lipase, pancreatic enzymes

## Abstract

**Introduction:**

Acute pancreatitis (AP) and diabetic ketoacidosis (DKA) can influence each other, and diagnosing AP in DKA patients is challenging due to overlapping symptoms and laboratory findings.

**Case Presentation:**

This case series discusses two adolescents with type 1 diabetes who presented with severe abdominal pain and were diagnosed with both DKA and AP. Both had a history of poor medication adherence. Initial labs showed hyperglycemia, acidosis, and elevated pancreatic enzymes. While DKA symptoms resolved quickly, abdominal pain persisted and imaging confirmed pancreatitis. Treatment included fluids, insulin, pain management, and symptoms management for pain as well as the use of antibiotics and total parenteral nutrition (TPN). Both patients improved gradually and were discharged with advice for better diabetes control.

**Conclusion:**

Diagnosing AP in DKA is difficult because of similar symptoms and laboratory findings. Imaging can help but is not always definitive. Unnecessary pancreatic enzyme testing and the use of antibiotics, opioid analgesia, and TPN treatments, if not clinically indicated, should be avoided.

## 1. Introduction

Diabetic ketoacidosis (DKA) is a prevalent complication associated with type 1 diabetes (T1D), typically diagnosed using standard criteria. It represents a significant cause of morbidity and mortality among T1D patients [1]. There exists a bidirectional cause-and-effect relationship between acute pancreatitis (AP) and DKA. Diagnosing AP in the context of DKA can be particularly challenging due to the overlap in clinical and laboratory features [2–5]. The pancreatic enzymes amylase and lipase may be elevated in one or both conditions when they coexist, further complicating the diagnostic process [3].

Managing suspected AP in children with DKA presents various challenges and pitfalls, as the two conditions share overlapping clinical and laboratory features and require special considerations in treatment. In 2018, the North American Society for Pediatric Gastroenterology, Hepatology and Nutrition (NASPGHAN) published the first English-language clinical report addressing the diagnostic and management challenges of pediatric AP; prior to this, no formal guidance existed in the field [6]. The primary aims of this clinical report are to review the published evidence on the management of AP in children, compare and contrast pediatric and adult literature, identify gaps and limitations in the existing knowledge, and provide recommendations to support a unified approach for clinicians managing children with AP [6].

There remains a lack of robust evidence in the field of pediatric pancreatitis, particularly concerning fluid management, pain control, dietary interventions, and consistency in follow-up care [7]. Although many questions remain in these areas, the pancreatitis working group of the British Society of Paediatric Gastroenterology, Hepatology and Nutrition (BSPGHAN) published guidance in 2020, which was updated in 2024 [7, 8]. This guidance is intended to provide a framework for multidisciplinary teams caring for young patients with AP, promoting a more standardized approach to their management. The management of suspected AP in hyperglycemic states necessitates careful diagnostic confirmation through imaging, effective pain management that minimizes opioid use, and avoidance of unnecessary antibiotic therapy [7–9]. It emphasizes the preferential use of enteral nutrition over total parenteral nutrition (TPN), alongside aggressive yet balanced fluid and insulin therapy aimed at resolving both pancreatitis and the metabolic crisis [7–9]. Additionally, close multidisciplinary monitoring and tailored treatment of complications are essential [7–9]. This comprehensive approach helps mitigate the risks and complexities associated with treating patients experiencing both AP and hyperglycemic emergencies.

This report presents a case that illustrates the overlap between AP and DKA, highlighting critical practical issues regarding the diagnosis and management of such cases.

## 2. Case Series

### 2.1. Case 1

We present a 14-year-old girl with T1D, diagnosed 7 years prior, who was started on insulin glargine at 20 units daily and insulin aspart at eight units three times daily. She had no comorbidities and was not taking any medications other than those prescribed for her T1D. However, her diabetes management had been suboptimal, necessitating multiple hospital admissions for DKA across various institutions. Four days postdischarge from another facility, she arrived at our emergency department with complaints of persistent vomiting, drowsiness, abdominal pain, polyuria, polydipsia, and tachypnea. Notably, she exhibited no fever or additional symptoms. Her mother indicated adherence to insulin therapy before the onset of her symptoms, although her most recent glycated hemoglobin (HbA1c) was recorded at 11.7%. Upon examination, the patient was tachycardic (heart rate = 160 bpm), appeared emaciated, and was moderately dehydrated. Her respiratory rate was 30 breaths per minute, and body temperature was 37.2°C. She maintained a hemoglobin oxygen saturation of 98% on 1.5 liters of oxygen via a nonrebreather mask. The abdominal examination revealed a soft, nontender abdomen with no rebound tenderness or organomegaly, and and no periumbilical or flank bruising were noted. The remainder of the systemic examination was unremarkable.

**Figure 1:**
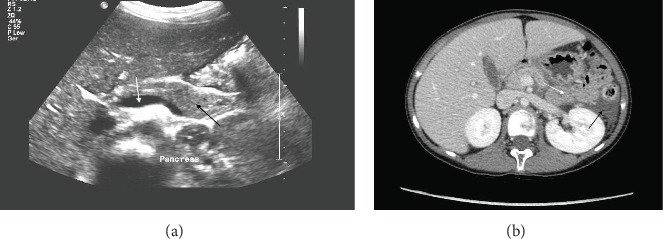
Ultrasound and CT scan changes in the pancreas in patient 1. (a) Ultrasound abdomen showed slightly enlarged pancreas (black arrow) with minute peri-pancreatic fluid (white arrow). (b) CT abdomen showed features consistent with acute pancreatitis including pancreatic tail swelling (white arrow), peri-pancreatic fluid tracking along the left para-colic gutter, small amount of fluid in the pelvis, and small left sided pleural effusion.

Diagnosis of DKA was established based on her clinical presentation, evident metabolic acidosis from blood gas analysis, hyperglycemia, and ketonuria and absence of other possible causes of metabolic acidosis. Of note, our patient also had a sodium level of 137 mmol/L and a glucose level of 38.9 mmol/L, resulting in a calculated effective serum osmolality of 313 mOsm/kg, which meets the criteria for HHS. The corrected sodium was 151 mmol/L, indicating hypernatremia. These findings confirmed the diagnosis of DKA/HHS, which guided the standardized approach to fluid and insulin management in this patient and contributed to a successful outcome. Ranitidine was also administered, and the patient was kept nil by mouth due to significant abdominal pain. Ceftriaxone was also started in the emergency room, although the rationale for this decision was inadequately documented. Despite the resolution of DKA within the first night of admission, her abdominal pain intensified, particularly with movement, but was alleviated by sitting and leaning forward. Vital signs remained stable, and physical examination revealed mild epigastric tenderness. Laboratory tests indicated elevated serum amylase (1186 U/L; normal 25–125) and lipase (5457 U/L; normal 0–200), accompanied by normal liver function tests. Further lab results showed urine ketone levels of + 4, which later normalized, and urine glucose levels of + 4, which also resolved. Her white blood cell count was elevated at 18 × 10^9^/L, decreasing to 10.9 × 10^9^/L by the first day, with neutrophils initially high at 12.9 × 10^9^/L, dropping to 7.6 × 10^9^/L. Hemoglobin was recorded at 16.5 g/dL, and HbA1c was 11.7% at presentation. Additionally, triglyceride levels measured on the second day were slightly elevated at 1.2 mmol/L. Morphine was administered in the pediatric intensive care unit (PICU) to manage her pain that did not settle with nonopioid analgesia, and TPN was initiated for 3 days until she could tolerate oral intake. An abdominal ultrasound revealed a slightly enlarged pancreas with minimal peri-pancreatic fluid. A computed tomography (CT) scan showed features consistent with AP, including swelling of the pancreatic tail, peri-pancreatic fluid tracking along the left para-colic gutter, a small amount of fluid in the pelvis, and a small left-sided pleural effusion (Figure 1). The liver and biliary system appeared unremarkable. Therefore, a diagnosis of acute no-biliary pancreatitis was established. Supportive care was continued until the pancreatic enzyme levels stabilized over the next 3 days (Table 1). The patient was discharged after a 7-day hospital stay without complications.

### 2.2. Case 2

A 15-year-old male with T1D, diagnosed 10 years prior, presented with severe abdominal pain, recurrent vomiting, rapid breathing, and significant dehydration, without fever or other systemic symptoms. His family reported poor adherence to medication before the onset of symptoms. He had no comorbidities and was not taking any medications other than those prescribed for her T1D. Physical examination revealed a lethargic, severely dehydrated, and tachycardic patient with a heart rate of 140 bpm. His respiratory rate was elevated at 28 breaths per minute, and body temperature was 37.5°C. Hemoglobin oxygen saturation was maintained at 98% on room air. The abdominal examination showed distension and mild tenderness in the epigastric region without rebound tenderness or organomegaly, and no periumbilical or flank bruising.

Initial laboratory investigations indicated a pH range of 7.166–7.371, a low bicarbonate level (10–19 mmol/L), slightly reduced serum sodium (128–135 mmol/L), and normal potassium levels (4.7–5.3 mmol/L). Serum urea and creatinine levels were 2.5–2.9 mmol/L and 76–100 µmol/L, respectively. The patient was hyperglycemic, with an HbA1c of 9.6%, fasting blood sugar levels ranging between 7.4 and 15.1 mmol/L, osmolality at 275–281 mosm/Kg, significant ketonuria (+3), and glucosuria (+4). His white blood cell count was elevated at 11.1 × 10^9^ cells/L, with neutrophils at 10.2 × 10^9^ cells/L, and hemoglobin at 14.7 g/dL. Elevated pancreatic enzymes were noted, with lipase at 3400 U/L and amylase at 375 U/L, suggesting AP (Table 2).

The initial diagnosis of DKA was based on hyperglycemia, acidosis on arterial blood gas, and significant ketonuria without any evident alternative causes of metabolic acidosis. Management followed standard DKA guidelines, including fluid resuscitation and insulin therapy. Due to severe epigastric pain, intravenous (IV) pantoprazole was administered. Additionally, a course of IV tazocin (3 gm) was started on the third day for a perianal abscess. Although DKA symptoms resolved within the first 24 h, abdominal pain persisted, described as constant and radiating to the back, with slight relief when leaning forward. Repeated physical examinations indicated ongoing epigastric tenderness. Laboratory tests on day four revealed elevated serum amylase (207 U/L) and lipase (2887 U/L). The patient was treated with analgesics in the PICU and placed on TPN for 5 days.

**Figure 2:**
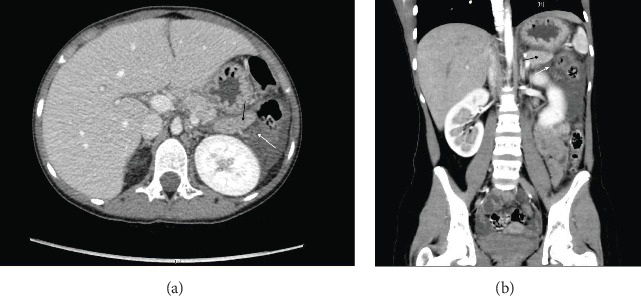
CT and MRI of pancreas with some changes in patient 2. (a) CT abdomen showed diffuse pancreatic enlargement (black arrow) and peri-pancreatic fluid (white arrow). (b) MRI abdomen showed diffuse pancreatic enlargement (black arrow) and peri-pancreatic fluid (white arrow).

While an abdominal ultrasound was inconclusive, CT and subsequent MRI scans confirmed findings consistent with AP, including diffuse pancreatic enlargement and extensive peri-pancreatic fluid, without evidence of necrosis or pseudocysts (Figure 2). The gallbladder and biliary tract appeared normal. Supportive care continued, and the patient showed gradual improvement, with normalization of pancreatic enzymes over 5 days. By the final assessment, his condition significantly improved: pH normalized to 7.475, bicarbonate increased to 26 mmol/L, in arterial blood gas and other electrolytes stabilized with sodium at 134 mmol/L and potassium at 3.9 mmol/L. Serum urea rose slightly to 3.2 mmol/L, and creatinine decreased to 47 µmol/L. Blood sugar levels stabilized between 6.4 and 13.6 mmol/L, and pancreatic enzymes significantly decreased, with lipase at 1177 U/L and amylase at 132 U/L, indicating resolution of pancreatitis (Table 2). He was discharged on the eighth day with recommendations for stricter glycemic control and dietary modifications to better manage his diabetes and lipid levels.

## 3. Discussion

The primary challenge in managing AP in children with DKA is the diagnostic overlap between the two conditions, especially since elevated pancreatic enzymes in DKA can lead to misdiagnosis. Diagnosing AP in the context of DKA is a complex challenge. Lipase and amylase levels can be elevated in 38% and 19% of DKA patients, respectively, and may exceed three times the normal range [4]. The elevation of these enzymes is believed to result from a leak from pancreatic acinar cells as well as from nonpancreatic sources (e.g., salivary, gastric, and hepatic) [3]. To minimize confusion caused by these common and nonspecific elevations of pancreatic enzymes in DKA, it is advised not to measure these enzymes unless there is a clinical suspicion of AP [4]. Abdominal pain is a crucial symptom in raising the suspicion of AP, particularly when it persists following recovery from the acidotic state. Abdominal pain with severe diabetic complications should prompt consideration of AP, especially since hypertriglyceridemia, a frequent finding in these states, can both cause and complicate pancreatitis, with impressive triglyceride levels sometimes exceeding 2000 mg/dL [10]. Therefore, if abdominal pain is absent, a diagnosis of AP should not be considered solely based on elevated pancreatic enzyme levels [3]. However, when abdominal pain is present alongside elevated pancreatic enzymes, it can still be nonspecific for AP in the setting of unresolved DKA, as DKA itself can produce both findings. This underscores the utility of CT scans, which can reveal changes consistent with AP in up to 80%–85% of cases [2, 3, 11, 12]. Conversely, the diagnosis may not be confirmed in 20%–25% of AP cases where CT scans appear unremarkable [2, 5]. In general, diagnosis relies on clinical assessment, amylase/lipase elevation, and careful pancreatic imaging, as metabolic disturbances may confound typical presentations [13, 14]. Additionally, the absence of significant abdominal pain in at least 11% of AP cases can further complicate the diagnosis of AP in DKA patients [3].

Similar to our patients, the coexistence of AP and DKA has been associated with more severe hyperglycemia, acidosis, and a wider anion gap. These patients are more likely to require admission to PICU; however, they do not exhibit differences in the length of PICU or hospital stay compared to DKA patients without AP [2]. It is essential to remember that in DKA patients, amylase is slightly more specific than lipase (97% vs. 91%), and lipase levels appear to correlate better with the severity of acidosis [2, 4]. Moreover, as in case 1, the diagnosis of DKA/HHS emphasizes the occasional complexity of the clinical scenario, alongside other issues such as infection (abscess and antibiotic use). This illustrates the challenges encountered in the clinical presentation and management of AP in the context of diabetes-related hyperglycemic complications. HHS, in particular, may have a higher mortality rate than DKA and could be associated with hypertriglyceridemia that can exacerbate AP although it was only mildly elevated in our patient [10].

Management requires careful balancing of fluid resuscitation, nutritional timing, and pain control, all of which must be tailored to the child's evolving clinical status. Early identification of hypertriglyceridemia as a potential trigger for AP in the context of DKA is also important for guiding appropriate treatment. Optimal care is best achieved through a multidisciplinary approach involving pediatric endocrinology, gastroenterology, and critical care teams. Management prioritizes aggressive fluid resuscitation, insulin therapy to correct hyperglycemia and reduce triglyceride levels, and electrolyte monitoring [15]. Pain control must be balanced to avoid opioid-related adverse effects, and empirical antibiotics are reserved for proven infections [13]. Nutritional support favors early enteral feeding over TPN to reduce complications such as worsening of and metabolic derangements [13]. Optimal outcomes depend on addressing precipitating factors, close monitoring of biochemical parameters, and multidisciplinary specialist involvement to coordinate insulin therapy, manage metabolic derangements, and prevent recurrence of DKA or pancreatitis [15, 16].

As illustrated in our cases, common management pitfalls may include the unnecessary use of antibiotics, morphine, and TPN. Antibiotics are not typically indicated empirically in either DKA or AP [17]. However, in case 2, tazocin (piperacillin-tazobactam) was administered for a perianal abscess, which is supported by literature for its broad-spectrum efficacy against both aerobic and anaerobic bacteria, making it suitable for complicated intra-abdominal infections, including those related to diabetic patients with soft tissue infections [18]. Meperidine (also called pethidine) has been recommended as an alternative to morphine in cases of AP, as the latter can increase the pressure at the sphincter of Oddi (SO), theoretically exacerbating pancreatitis and potentially increasing the risk of cholecystitis, although the literature shows no significant differences regarding these outcomes [19]. Some studies indicate that narcotics can elevate biliary pressure and disrupt SO peristalsis, possibly worsening pain in AP [19]. Although some pediatric gastroenterologists or surgeons may still occasionally prescribe meperidine for AP pain, current guidelines and evidence recommend minimizing its use in children [20, 21]. Safer and more effective alternatives, such as morphine, are now preferred for pain management in these cases [20, 21].

Furthermore, initiating TPN in AP patients is generally unjustified, as controlled trials have consistently demonstrated higher rates of mortality, multiple organ failure, and systemic infections associated with this treatment in such scenarios [22, 23]. It has also been shown to prolong the need for renal replacement therapy when necessary, in contrast to enteral feeding. Instead, it is recommended to provide nutrition to patients unable to tolerate oral intake via a naso-jejunal tube with a semi-elemental formula that is high in protein and low in fat [22, 23]. Overall, TPN is almost never indicated in the management of DKA in children and should not be considered a standard treatment [24]. In cases of AP, TPN is considered a last-resort option and is only initiated when enteral nutrition is not possible for a prolonged period, typically 5–7 days, in complicated cases involving gastrointestinal obstruction, ileus, or other factors that prevent safe enteral access, or when caloric requirements cannot be met through oral or enteral routes [25]. For both AP and poststabilization DKA in pediatric patients, early enteral nutrition remains the standard of care [6, 7]. Last but not least, vitamin K is a fat-soluble vitamin needed for clotting factor synthesis. In chronic pancreatic disease with exocrine insufficiency, fat-soluble vitamin malabsorption can cause vitamin K deficiency over time [26]. However, during AP and/or DKA, vitamin K deficiency is uncommon and supplementation is rarely needed unless there is clear coagulopathy or bleeding [6]. Routine vitamin K administration is not recommended in pediatric pancreatitis or DKA guidelines, which focus on fluid, electrolyte, insulin, pain control, and nutritional support [6].

## 4. Conclusion

AP in the context of DKA poses diagnostic challenges due to the overlap between the two conditions in the clinical and metabolic features. While imaging is sensitive and informative, it is not definitive for diagnosing AP in DKA patients. Routine measurement of pancreatic enzymes without clinical indication such as severe abdominal pain is not advisable, as it can lead to unnecessary interventions. Treating physicians should exercise caution regarding the use of antibiotics, TPN, and analgesics in patients with AP and DKA.

## Figures and Tables

**Table 1:** Investigations of patient 1 during hospital admission.

Investigations	At presentation	Day 1	Day 2	Day 3	Day 4	Day 5	Day 6	Day 7	Day 21
pH	6.99	—	—	7.083	—	—	—	—	—
Bicarbonate	5.1	20.1	24.8	28.5	27.2	—	—	—	—
Serum sodium (mmol/L)	137	151	141	138	139	—	—	—	—
Serum potassium (mmol/L)	4.7	3.4	3.5	3.9	3.9	—	—	—	—
Serum urea (mmol/L)	10.2	3.9	2.3	2.7	2.6	—	—	—	—
Serum creatinin (mmol/L)	111	48	43	41	37	—	—	—	—
Random blood sugar (mmol/L)	38.9	8.2	11	13.9	11.1	—	—	—	—
Lipase (normal 0–200 U/L)	—	5457	3444	2200	1334	—	1862	1211	—
Amylase (normal 25–125 U/L)	—	1186	588	272	210	—	190	177	—
Treatment 1: DKA protocol or S/C insulin	IV fluids and insulin infusion	IV fluids and insulin infusion	IV fluids and insulin infusion	IV fluids and insulin infusion	IV fluids and insulin infusion	Glargine and aspart insulins	Glargine and aspart insulins	Glargine and aspart insulins	Glargine and aspart insulins
Treatment 2	Paracetamol for abdominal pain and granisetron	Paracetamol and IV ranitidine	TPN IV ranitidine	TPN morphine for abdominal pain	TPN	TPN	—	—	—

Abbreviations: DKA, diabetic ketoacidosis; HBA1c, glycated hemoglobin; IV, intravenous; S/C, subcutaneous; TPN, total parenteral nutrition; WBC, white blood cell count.

**Table 2:** Investigations of patient 2 during hospital admission.

Investigation/day	At presentation	Day 1	Day 2	Day 3	Day 4	Day 5	Day 6	Day 7
pH	7.166–7.371	7.344–7.38	7.379–7.393	7.475	—	—	—	—
Bicarbonate (mmol/L)	10–19	19–20	20–23	26	—	—	—	—
Serum sodium (mmol/L)	128–135	134–137	135	134	—	—	—	—
Serum potassium (mmol/L)	4.7–5.3	3.5–4.6	3.2	3.9	—	—	—	—
Serum urea (mmol/L)	2.5–2.9	0.9–2.4	1.3	3.2	—	—	—	—
Serum creatinine	76–100	47–67	50	47	—	—	—	—
Random blood sugar (mmol/L)	Fasting BS	Fasting BS	Fasting BS	6.9–8	2.4–15.7	6.1–20.1	4.1–27.4	6.4–13.6
	7.4–15.1	6.6–12.4	6.9–13.4	2 Readings only				
Serum osmolality (mosm/Kg)	275–281	270–274	274	269	—	—	—	—
Lipase (U/L)	3400	3930	2211	1814	2887	—	3080	1177
Amylase (U/L)	375	452	207	303	207	—	226	132
IV fluids with potassium and insulin infusion (DKA protocol)	Yes	Yes	Glargine and aspart were resumed	Glargine and aspart were resumed	Glargine and aspart were resumed	Glargine and aspart were resumed	Glargine and aspart were resumed	Glargine and aspart were resumed
Treatment	Phytonadione (vitamin K) IV 5 mg (1 dose)	Phytonadione (vitamin K) IV 5 mg (1 dose)	Phytonadione (vitamin K) IV 5 mg (1 dose)	Pantoprazol IV 40 mg (2 doses)	Pantoprazol IV 40 mg (2 doses)	Pantoprazol IV 40 mg (1 dose)	—	—
	Ranitidine IV 40 mg (once)	Paracetamol IV 500 mg (2 doses)	Morphine IV 2 mg (1 dose)			Paracetamol IV 500 mg (1 dose)	—	—
	Pantoprazol+NaCl 0.9%	Pantoprazol+NaCl 0.9%	Pantoprazol IV 40 mg				—	—

Abbreviations: DKA, diabetic ketoacidosis; HBA1c, glycated hemoglobin; IV, intravenous; TPN, total parenteral nutrition; WBC, white blood cell count.

## Data Availability

The data that support the findings of this study are available on request from the corresponding author. The data are not publicly available due to privacy or ethical restrictions.

## Nomenclature

AP: Acute pancreatitis
CT: Computed tomography
DKA: Diabetic ketoacidosis
HbA1c: glycated hemoglobin
PICU: Pediatric intensive care unit
SO: Sphincter of Oddi
TPN: Total parenteral nutrition
WBC: White blood cell count.
